# Free-space high-Q nanophotonics

**DOI:** 10.1038/s41377-025-01825-x

**Published:** 2025-04-27

**Authors:** Jianbo Yu, Wenzhe Yao, Min Qiu, Qiang Li

**Affiliations:** 1 State Key Laboratory of Extreme Photonics and Instrumentation, College of Optical Science and Engineering, Zhejiang University, Hangzhou, 310027 China; 2 Key Laboratory of 3D Micro/Nano Fabrication and Characterization of Zhejiang Province, School of Engineering, Westlake University, Hangzhou, 310024 China

**Keywords:** Nanophotonics and plasmonics, Metamaterials

## Abstract

High-Q nanophotonic devices hold great importance in both fundamental research and engineering applications. Their ability to provide high spectral resolution and enhanced light-matter interactions makes them promising in various fields such as sensing, filters, lasing, nonlinear optics, photodetection, coherent thermal emission, and laser stealth. While Q-factors as large as 10^9^ have been achieved experimentally in on-chip microresonators, these modes are excited through near-field coupling of optical fibers. Exciting high-Q modes via free-space light presents a significant challenge primarily due to the larger fabrication area and more lossy channels associated with free-space nanophotonic devices. This Review provides a comprehensive overview of the methods employed to achieve high-Q modes, highlights recent research progress and applications, and discusses the existing challenges as well as the prospects in the field of free-space high-Q nanophotonics.

## Abstract (web summary)

This article presents a comprehensive overview of free-space high-Q nanophotonics, encompassing its physical principles, recent advancements, and various applications.

## Graphical abstract

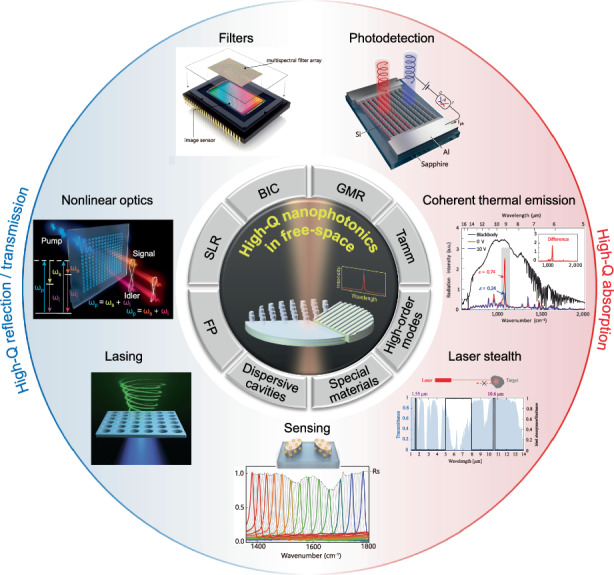

## Introduction

**Fig. 1:**
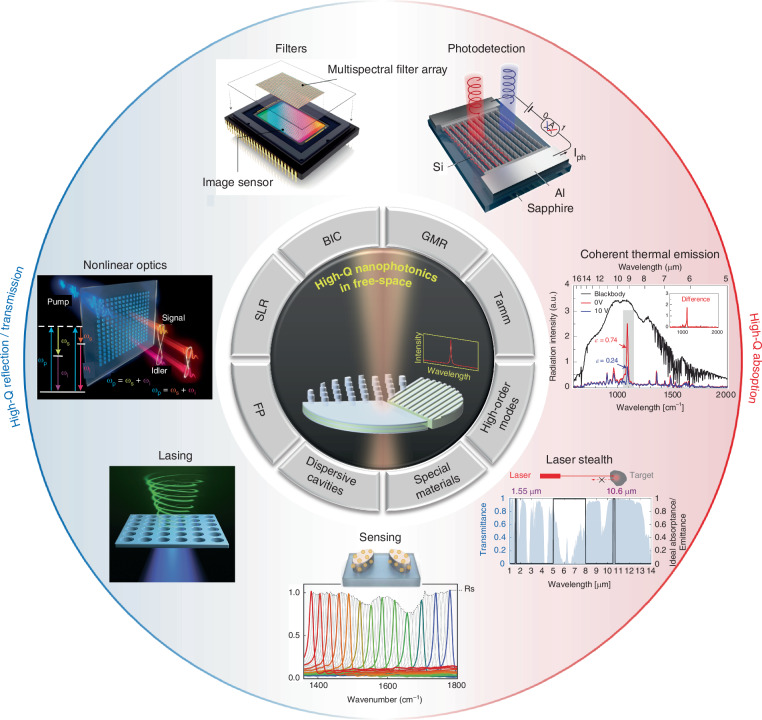
Schematic of free-space high-Q nanophotonic devices and their relevant applications including sensing^4^, filters^5^, lasing^6^, nonlinear optics^7^, photodetection^8^, coherent thermal emission^9^ and laser stealth^10^. Panels adapted from: AAAS^4,6,7^; ACS^5^; Optica^8^; Springer Nature^9^; Elsevier^10^

The fast development of nanophotonics has opened up new possibilities to manipulate the spectrum, polarization and phase of light at subwavelength scale, thereby facilitating the miniaturization and integration of optical devices^1–3^. High-Q (quality factor) nanophotonics, in particular, has emerged as a promising area of investigation. High-Q modes endow optical devices with higher spectral resolution, greater sensitivity, faster modulation speed, and stronger light-mater interactions. Consequently, they have attracted considerable interest for a wide range of applications such as sensing^4^, filters^5^, lasing^6^, nonlinear optics^7^, photodetection^8^, coherent thermal emission^9^ and laser stealth^10^, as illustrated in Fig. 1.

While experimental achievements have demonstrated Q-factors as high as 10^9^ in on-chip microresonators, these modes typically require excitation through near-field coupling from optical fibers^11^. It is still challenging to excite high-Q modes via free-space light (i.e., propagating light lying above the air light cone). This primarily lies in the fact that most of the free-space nanophotonic devices consist of nanostructure arrays with larger areas compared with a single microresonator, which can result in more fabrication imperfections. These fabrication imperfections not only degrade the quality of individual resonators, leading to a decrease in the overall Q-factor, but also allow coupling between modes at different points in k-space, leading to additional lossy channels into free-space and further reducing the Q-factor^12^. Nowadays, with the deployment of 5 G network and development of 6 G technology, free-space nanophotonic devices are gaining increasing attention due to their potential applications in augmented reality (AR), virtual reality (VR) devices and high-speed wireless communication^13^. Therefore, it is crucial to analyze the underlying physical principles behind achieving high-Q modes in free-space nanophotonic devices and explore the methods to improve Q-factors in experiments. These efforts hold significant importance for the design of optoelectronic devices with novel and superior functionalities.

In this Review, we summarize the basic physical principles involved in achieving high-Q modes and recent advancements in high-Q nanophotonic devices excited by free-space light. We begin by analyzing the spectral characteristic of an optical resonator using temporal coupled-mode theory (TCMT). This theory serves as a foundation for understanding the resonance behavior of high-Q modes. Next, we show the underlying physical mechanisms for various methods of achieving high-Q modes. Then, we review the recent research progress on high-Q non-absorbing and absorbing nanophotonic devices, along with their respective applications. Finally, we summarize the existing challenges and discuss the prospects for further advancements in the field.

## Principle

Q-factor is a parameter to describe the damping of a mode, which is defined as the ratio of the total stored energy to the energy lost during one time-oscillation cycle of the mode. To distinguish the contributions of radiative and non-radiative decay to the energy loss process, the total Q-factor *Q*_tot_ is expressed as ${{Q}_{{\rm{tot}}}}^{-1}={{Q}_{{\rm{r}}}}^{-1}+{{Q}_{{\rm{n}}}}^{-1}$, where *Q*_r_ and *Q*_n_ are the radiative and non-radiative Q-factor, respectively. With the well-defined Q-factors, the spectral characteristic of an optical resonator can be well analyzed.

A high-Q mode requires simultaneously high *Q*_r_ and *Q*_n_. A high *Q*_r_ suggests that the energy leaks into the environment slowly, while a high *Q*_n_ indicates that the energy decay inside the resonator (e.g., due to absorption) is slow. To increase these Q-factors, precisely engineering the shape, size, and material of the resonator is required.

### Theoretical analysis of optical resonators

We analyze the modes in an optical resonator via TCMT. TCMT can be applied to resonators consisting of several modes weakly coupled to each other and to a given number of ports^14^. Suppose the electromagnetic field varies in time as ${e}^{-i\omega t}$, the radiative and nonradiative decay rate of a mode can be expressed as ${\gamma }_{{\rm{r}}}={\omega }_{0}/(2{Q}_{{\rm{r}}})$ and ${\gamma }_{{\rm{n}}}={\omega }_{0}/(2{Q}_{{\rm{n}}})$, respectively, where ${\omega }_{0}$ is the resonant frequency. The dynamic equations for the amplitudes of all modes in the resonator are

1 $$\frac{{\rm{d}}{\bf{a}}}{{\rm{d}}t}=\left(-i\varOmega -{\varGamma }_{{\rm{r}}}-{\varGamma }_{{\rm{n}}}\right){\bf{a}}+{K}^{{\rm{T}}}{{\bf{s}}}_{+}$$

2 $${{\bf{s}}}_{-}=C{{\bf{s}}}_{+}+D{\bf{a}}$$

where **a** is the normalized amplitude vector, so that ${|{a}_{{\rm{i}}}|}^{2}$ corresponds to the energy of the *i*th mode inside the resonator. **s**_+_ and **s**_−_ are the amplitude vectors of incoming and outgoing waves, respectively. *Ω* represents resonant frequencies (diagonal elements) and near-field coupling coefficients between different modes (off-diagonal elements), *Γ*_r_ represents the radiative decay rates (diagonal elements) and far-field coupling coefficients between different modes (off-diagonal elements), *Γ*_n_ represents the non-radiative decay rates, *K*^T^ is the coupling matrix from the incoming waves to modes, *D* is the coupling matrix from modes to outgoing waves, *C* is the background scattering matrix, through which the incoming and outgoing waves can couple directly. For lossless and reciprocal system, the matrix *C* is unitary and symmetric^15,16^. In lossless systems, the matrix *K*^T^, *D*, and *C* are related to each other due to the energy conservation and time-reversal symmetry as

3 $$K=D$$

4 $${D}^{\dagger }D=2{\varGamma }_{{\rm{r}}}$$

5 $$C{D}^{* }=-D$$

In lossy systems, Eqs. (3)–(5) remains valid under the assumption that the introduction of intrinsic loss does not change the coupling coefficients among different modes and ports^17,18^. TCMT is valid when (1) The modes have high Q-factors (${\gamma }_{{\rm{r}}}+{\gamma }_{{\rm{n}}}\ll {\omega }_{0}$) so that the coupling coefficients can be taken to be frequency independent^19^. (2) All modes weakly couple to each other (i.e., the coupling coefficient is much less than the resonant frequencies) so that the coupling affects the amplitudes only when two modes have similar resonant frequencies and the derivative of **a** is approximately equal to −*iω***a**^20^. The scattering matrix *S* of the resonator is given by

6 $$S=C+D{\left[-i\left(\omega I-\varOmega \right)+{\varGamma }_{{\rm{r}}}+{\varGamma }_{{\rm{n}}}\right]}^{-1}{K}^{{\rm{T}}}$$

where *I* is the identity matrix. From Eq. (6), the reflection, transmission and absorption spectra can be obtained. In the following, we demonstrate the calculated spectra of one-mode and two-mode resonators according to TCMT. The theoretical model can be used to analyze most of the photonic systems and wave phenomenon such as Fano line shape, electromagnetically induced transparency (EIT), and perfect absorption, etc.

#### One-mode resonators

Consider a two-port optical resonator supporting one mode with the resonant frequency *ω*_0_ and non-radiative decay rate *γ*_n_. The mode can decay to Port 1 and Port 2 with the radiative decay rates *γ*_r1_ and *γ*_r2_, respectively. Light is incident from Port 1, and the background reflection and transmission coefficient are *r*_b_ and *t*_b_, respectively. From the scattering matrix, the reflection, transmission, and absorption are expressed as

7 $$R\left(\omega \right)=\frac{{\left[\left|{r}_{{\rm{b}}}\right|\left(\omega -{\omega }_{0}\right)\pm \left|{t}_{{\rm{b}}}\right|{\sigma }_{1}\right]}^{2}+{\left({\sigma }_{2}+\left|{r}_{{\rm{b}}}\right|{\gamma }_{{\rm{n}}}\right)}^{2}}{{\left(\omega -{\omega }_{0}\right)}^{2}+{\left({\gamma }_{{\rm{r}}1}+{\gamma }_{{\rm{r}}2}+{\gamma }_{{\rm{n}}}\right)}^{2}}$$

8 $$T\left(\omega \right)=\frac{{\left[\left|{t}_{{\rm{b}}}\right|\left(\omega -{\omega }_{0}\right)\mp \left|{r}_{{\rm{b}}}\right|{\sigma }_{1}\right]}^{2}+{\left(\left|{t}_{{\rm{b}}}\right|{\gamma }_{{\rm{n}}}\right)}^{2}}{{\left(\omega -{\omega }_{0}\right)}^{2}+{\left({\gamma }_{{\rm{r}}1}+{\gamma }_{{\rm{r}}2}+{\gamma }_{{\rm{n}}}\right)}^{2}}$$

9 $$A\left(\omega \right)=\frac{4{\gamma }_{{\rm{r}}1}{\gamma }_{{\rm{n}}}}{{\left(\omega -{\omega }_{0}\right)}^{2}+{\left({\gamma }_{{\rm{r}}1}+{\gamma }_{{\rm{r}}2}+{\gamma }_{{\rm{n}}}\right)}^{2}}$$

1. when $|{r}_{{\rm{b}}}|=0$, $|{t}_{{\rm{b}}}|=1$, we have ${\sigma }_{1}=\sqrt{2{{\gamma }_{{\rm{r}}1}}^{2}+2{{\gamma }_{{\rm{r}}2}}^{2}}$ and ${\sigma }_{2}=0$.
2. when $|{r}_{{\rm{b}}}|=1$, $|{t}_{{\rm{b}}}|=0$, we have ${\sigma }_{1}=0$ and ${\sigma }_{2}={\gamma }_{{\rm{r}}2}-{\gamma }_{{\rm{r}}1}$.
3. when $0 \,< \,|{r}_{{\rm{b}}}| \,< \,1$, $0\, < \,|{t}_{{\rm{b}}}|\, < \,1$, we have ${\sigma }_{1}=\frac{\sqrt{2{{\gamma }_{{\rm{r}}1}}^{2}+2{{\gamma }_{{\rm{r}}2}}^{2}-{|{r}_{{\rm{b}}}|}^{2}{\left({\gamma }_{{\rm{r}}2}+{\gamma }_{{\rm{r}}1}\right)}^{2}-{\left({\gamma }_{{\rm{r}}2}-{\gamma }_{{\rm{r}}1}\right)}^{2}/{|{r}_{{\rm{b}}}|}^{2}}}{|{t}_{{\rm{b}}}|}$, and ${\sigma }_{2}=\frac{{\gamma }_{{\rm{r}}2}-{\gamma }_{{\rm{r}}1}}{|{t}_{{\rm{b}}}|}$.

As can be seen, the reflection and transmission can have an asymmetric Fano line shape as long as the background reflection coefficient ${r}_{{\rm{b}}}$ is non-zero. This is because of the interference between the mode and the background. In contrast, the absorption is independent on the background reflection and transmission coefficients and always has a Lorentz line shape.

From a physical point of view, the radiative decay rates ${\gamma }_{{\rm{r}}1}$ and ${\gamma }_{{\rm{r}}2}$ are dependent on the symmetric properties of the resonator.

1. When the resonator has mirror symmetry with respect to the middle plane (e.g., a single layer of free-standing metasurface), the mode decays to the two ports with the same rates: ${\gamma }_{{\rm{r}}1}={\gamma }_{{\rm{r}}2}$. At the resonance frequency of ${\omega }_{0}$, the absorptance reaches maximum of ${A}_{\max }=50 \%$ when ${\gamma }_{{\rm{r}}1}+{\gamma }_{{\rm{r}}2}={\gamma }_{{\rm{n}}}$, which is known as critical coupling condition. For the reflection and transmission, they can have Fano line shapes when the mode interferences with the background, see Fig. 2a.

**Fig. 2:**
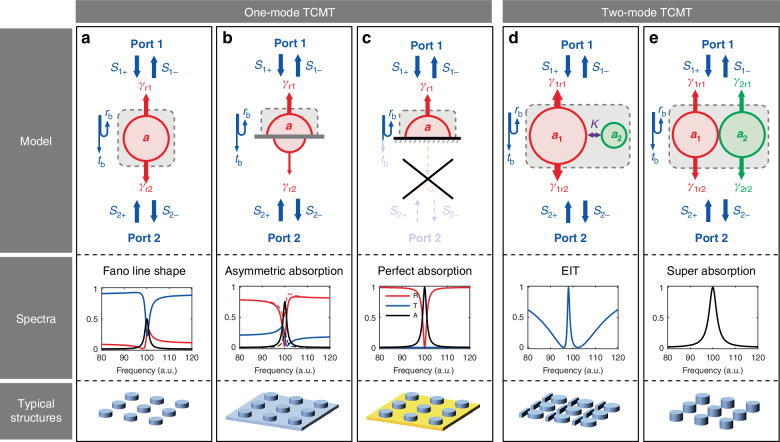
Analytical models of optical resonators. **a** One-mode resonators with the same radiative decay rates with respect to two ports (${\gamma }_{{\rm{r}}1}={\gamma }_{{\rm{r}}2}$). The maximum absorptance is 50%, and the reflection or transmission can have Fano line shape due to the interference between the mode and the background. **b** One-mode resonators with different radiative decay rates with respect to two ports (${\gamma }_{{\rm{r}}1}\,\ne \,{\gamma }_{{\rm{r}}2}$) due to structure asymmetry. The absorption of light incident from Port 1 (solid line) and Port 2 (dashed line) are asymmetric. **c** One-mode resonators with a reflective background that prohibits the transmission of light (${\gamma }_{{\rm{r}}2}=0$). Perfect absorption can be achieved when light is incident from Port 1. **d** Two-mode resonators where a bright mode with a large radiative decay rate couples with a dark mode with a negligible radiative decay rate. EIT can happen due to the mode coupling. **e** Two-mode resonators where two modes with different symmetry properties reside in the same resonator. Super absorption with the maximum absorptance larger than 50% can be achieved. For convenience, in (**d**) and (**e**), resonators with mirror symmetry with respect to the middle plane are chosen (${\gamma }_{{\rm{r}}1}={\gamma }_{{\rm{r}}2}$)
2. When the mirror symmetry is broken (e.g., a layer of metasurface backed by a thin dielectric film), the mode can decay to the two ports with different rates: ${\gamma }_{{\rm{r}}1}\,\ne \,{\gamma }_{{\rm{r}}2}$. Consider the time-reversal symmetry, the external light can couple with the mode with distinct coupling efficiency when incident from different ports. As a result, asymmetric absorption can be achieved, where the maximum absorptance is larger than 50% when light is incident from one port, and less than 50% when light is incident from the other port, see Fig. 2b.
3. When the transmission port is blocked (e.g., a layer of metasurface backed by an optically thick metal film), neither the mode can decay to Port 2 (${\gamma }_{{\rm{r}}2}=0$) nor the incident light can transmit directly to Port 2 (${t}_{{\rm{b}}}=0$). Perfect absorption can be achieved at the critical coupling condition (${\gamma }_{{\rm{r}}1}={\gamma }_{{\rm{n}}}$) when light is incident from Port 1. Away from the resonance frequency, light will be reflected, see Fig. 2c.

#### Two-mode resonators

When two modes exist in one resonator, they not only interact with the incident field but also influence each other through near-field mode overlapping, known as near-field coupling, or far-field interference, referred to as far-field coupling. The interplay between the two modes can give rise to new phenomena, such as EIT and super absorption.

1. Electromagnetically induced transparency
  EIT is a phenomenon arising from quantum interference, which leads to the emergence of a narrowband transparency window when light propagates through an originally opaque medium. The classical analogue of EIT in nanophotonics relies on the interference between a bright mode and a dark mode. When excited by certain types of free-space propagating light (e.g., plane waves), the bright mode can couple to the incidences directly over a broadband range. While the dark mode cannot be directly excited by the incidences^21^. The near-field coupling enables the dark mode to be excited by the energy scattered from the bright mode, resulting in the formation of a transparent window in the spectral response. Due to the negligible radiative decay rate of the dark mode, the transparent peak can be extremely sharp, see Fig. 2d.
2. Super absorption
  As has been discussed before, the maximum absorptance achievable for a freestanding single-mode metasurface is 50%. Though incorporating a metallic mirror behind the metasurface can increase the maximum absorptance to 100%, this approach results in the blocking of transmission at other frequencies, which limits its further applications. To achieve super absorption (${A}_{\max }\, > \,50 \%$) in a freestanding single-layer of metasurfaces, one can utilize the superposition of two modes with different symmetry. In this way, the maximum absorptance can reach 100% at the frequency degenerate point, see Fig. 2e. A typical structure employed to achieve super absorption is a dielectric metasurface that simultaneously supports electric- and magnetic-type Mie modes^22^.

### Physical mechanisms for high-Q modes

Achieving high-Q modes requires decreasing the radiative and non-radiative loss simultaneously. To reduce the radiative loss, bound states in the continuum (BICs), guided mode resonances (GMRs), surface lattice resonances (SLRs), high-order resonances, Tamm plasmon polaritons, Fabry-Perot (FP) resonances, and dispersion-assisted high-Q resonances can be utilized. To reduce the nonradiative loss, specific materials such as superconductor or gain materials can be utilized.

#### Bound states in the continuum

BICs represent the modes lying inside a continuous radiative spectrum but have zero radiative decay rates to the radiative channels^23^. Within the scope of free-space light excitation, BICs refer to the localized states lying in the continuous spectrum of radiative modes (modes above the light cone, such as free-space plane waves) but cannot couple to these radiative modes, and thus do not radiate energy away. BICs can be classified primarily into three categories based on the underlying physics mechanisms - Symmetry protected BICs, which refer to the bound states that belong to one symmetry class but are embedded within the continuous spectrum of another symmetry class^24^; Friedrich-Wintgen BICs (FW-BICs), which represent the mode with suppressed radiative loss due to the destructive interference with another mode residing at the same location^25–27^; Fabry-Perot BICs (FP-BICs), which represent the trapped modes between two ideal reflectors when the round-trip phase shift in the resonator equals an integer multiple of 2*π*^28,29^.

**Fig. 3:**
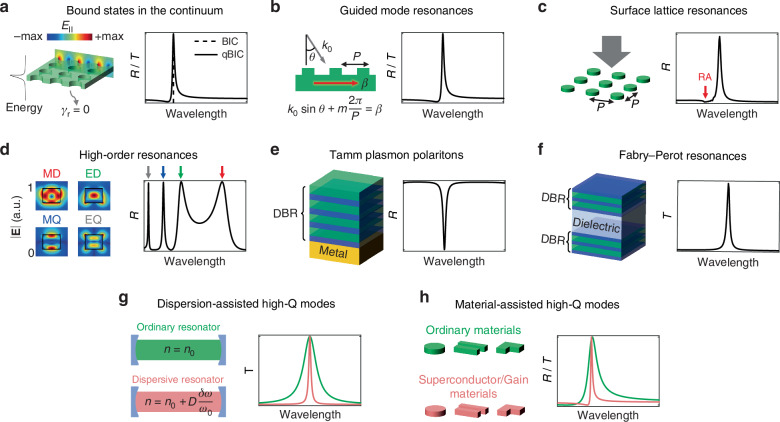
Methods of achieving high-Q modes. **a** Bound states in the continuum. **b** Guided mode resonances. **c** Surface lattice resonances. RA represents Rayleigh anomalies. **d** High-order resonances. **e** Tamm plasmon polaritons. **f** Fabry-Perot resonances. **g** Dispersion-assisted high-Q modes. **h** Material-assisted high-Q modes such as superconductor or gain materials

Theoretically, a BIC possesses an infinite radiative Q-factor and only exists in lossless and infinite structures^12^. An ideal BIC exhibits a zero radiative decay rate and cannot be excited by external incidence, see the dashed line in Fig. 3a. However, in practical applications, we often deal with quasi-BICs (qBICs) that have small but non-zero radiative decay rates. For example, symmetry protected BICs can be transformed into qBICs by introducing geometric or permittivity perturbation to break the symmetry^30^. QBICs are also known as supercavity modes, which exhibit finite but extremely high radiative Q-factors, and allow for external light excitation, as depicted by the solid line in Fig. 3a.

#### Guided mode resonances

GMRs are leaky guided surface modes. The dispersion lines of guided modes typically lie below the light cone, making the direct excitation by free-space light impossible. However, an additional layer of periodic shallow grating can compensate for the lateral wavenumber mismatch, and enables the coupling of free-space light with the guided modes. GMRs thereby exhibit a high sensitivity to both the incident angle and wavelength of external excitations^31^. Take the 1D periodic structures as an example, in order to excite the GMRs, the input angle ${\theta }_{{\rm{in}}}$ should satisfy the grating diffraction equation as ${k}_{0}\sin {\theta }_{{\rm{in}}}+m\frac{2\pi }{P}=\beta$, where ${k}_{0}$ is the wavenumber in vacuum, $m$ is an integer, $P$ is the grating period, and $\beta$ is the wavenumber of the guided mode.

GMRs can be achieved by combining a thin-film waveguide with a shallow grating. The high coherence exhibited by the nonlocal guided modes in the waveguide contributes to the high Q-factors observed in GMRs. In the absence of material absorption, the output spectra can have either a reflection or a transmission peak, characterized by Fano line shapes, see Fig. 3b. This spectral behavior arises from the interference between the GMRs and the thin film, which exhibits a non-zero background reflection coefficient. It should be noted that GMRs rely on the guided modes propagating along gratings, which sets them apart from high contrast gratings, where vertical Bloch modes are present between the upper and lower grating boundaries^31^.

#### Surface lattice resonances

SLRs originate from the far-field radiative coupling of the localized modes in individual nanoresonators, which can be observed in periodic nanophotonic structures. By carefully designing the resonant mode and the period, the scattered light from each nanoresonator can be in phase with the light scattered by adjacent ones. This results in a significant increase in the Q-factor of the mode, as the scattered light from neighboring nanoresonators can counteract the radiative decay of the individual nanoresonator^32^. As SLRs rely on collective resonances in periodic structures, their mode profiles can extend laterally over several unit cells, which can increase spatial coherence of electromagnetic waves. This make SLRs a good candidate for applications such as enhancing light emission from dye molecules^33^ or waveguides^34^. The radiative coupling of localized modes is enhanced by diffraction orders in the plane of the nanostructures, known as Rayleigh anomalies, making SLRs highly sensitive to both the period and the incident angle. Under normal incidence, SLRs typically occur when the wavelength equals to the period, accompanied by the Rayleigh anomalies in the spectrum (refer to Fig. 3c).

#### High-order resonances

The number of modes supported by a nanoresonator depends on its size. For example, dielectric metasurfaces with a high refractive index can support multiple Mie resonance modes. At longer wavelengths, low-order modes such as magnetic dipole (MD) and electric dipole (ED) can be observed, typically characterized by relatively low Q-factors (usually around ten^35^). As the wavelength decreases, multipole modes such as magnetic quadrupole (MQ) and electric quadrupole (EQ) become prominent. These multipole modes exhibit weaker radiative decay rates compared to dipole modes. Consequently, high-order resonances can be leveraged to achieve high Q-factors (one or two orders higher than low-order modes^36^) due to the reduced radiative loss and weaker coupling with incident light^37^, see Fig. 3d.

#### Tamm plasmon polaritons

Tamm plasmon polaritons state can be formed at the interface between a metal film and a dielectric Bragg mirror. The metal film exhibits near-unity reflection at frequencies well below the plasma frequency, and the Bragg mirror consisting of alternating layers of high and low refractive index materials reflects light strongly within the photonic stop band. This combination of high reflection from the metal and Bragg mirror allows for the formation of an eigenstate characterized by a small radiative decay rate and a high Q-factor, as shown in Fig. 3e. It is important to note that, unlike propagating surface plasmon polaritons (SPPs) that cannot be directly excited by incident light from the air due to their large wave vectors, Tamm plasmon polaritons possess an in-plane wave vector of zero and can be produced by direct optical excitation. Additionally, they can be excited by both transverse electric (TE) and transverse magnetic (TM) incident waves^38^.

#### Fabry-Perot resonances

FP resonance occurs when the round-trip phase shift experienced by light inside an optical cavity is an integer multiple of $2\pi$. A typical FP cavity consists of two parallel mirrors spaced by a certain distance, e.g., a dielectric film with a distributed Bragg reflector (DBR) on each side, see Fig. 3f. At the resonant frequencies, constructive interference occurs between the multiple reflections of light within the cavity, which leads to high transmission of light when the whole structure is lossless. The Q-factor of an FP resonance is dependent on the order of interference as well as the reflection of the two mirrors. Suppose the two mirrors have the same reflection $R$, the Q-factor of the *m*th order of FP resonance can be expressed as $Q=m\pi \sqrt{R}/\left(1-R\right)$. By increasing the resonance order (e.g., through increasing the cavity length) or the reflection of the mirror, the Q-factor of an FP resonance can be increased. When the materials constitute the FP cavity exhibit intrinsic loss, the transmission will decrease while the absorption will increase at the resonant frequencies^39^. It is noted that both FP resonances and GMRs mentioned in Section “Guided mode resonances” can be seen as the eigenmode solutions of source-free Maxwell’s equations in layered film structures. However, FP resonances are dependent on the vertical round-trip phase accumulation in the dielectric layers. They lie above the light-cone and be excited directly by free-space propagating light without additional scatterers. While GMRs rely on k-vectors of waveguide modes that propagating along the surface, and require additional scatterers such as gratings to bring them into light-cone, as discussed in Section “Guided mode resonances”.

#### Dispersion-assisted high-Q resonances

The Q-factor of a mode can be increased by engineering the dispersion of the resonator. Taking the FP cavity as an example, the frequency-dependent round-trip phase shift is expressed as ${\varphi }_{{\rm{nd}}}\left(\omega \right)=2{nL}{\omega }_{0}/{c}_{0}+2{nL}\delta \omega /{c}_{0}$, where ${nL}$ is the optical length of the cavity, ${c}_{0}$ is the vacuum light speed, ${\omega }_{0}$ is the resonant frequency, and the frequency detuning $\delta \omega =\omega -{\omega }_{0}$. Now suppose the cavity is made of the material with a frequency-dependent refractive index $n+D\delta \omega /{\omega }_{0}$, the round-trip phase shift is ${\varphi }_{{\rm{d}}}\left(\omega \right)\approx 2{nL}{\omega }_{0}/{c}_{0}+2(n+D)L\delta \omega /{c}_{0}$. The transmission spectra decrease to half of the maximum at the phase shift ${\varphi }_{{\rm{FWHM}}1}$ and ${\varphi }_{{\rm{FWHM}}2}$, respectively, and $\Delta \varphi ={\varphi }_{{\rm{FWHM}}2}-{\varphi }_{{\rm{FWHM}}1}$. For the non-dispersive resonator, the bandwidth is $\Delta {\omega }_{{\rm{nd}}}=\Delta \varphi /\left(2{n}_{0}L/{c}_{0}\right)$. While for the dispersive resonator, $\Delta {\omega }_{{\rm{d}}}=\Delta \varphi /\left[2\left({n}_{0}+D\right)L/{c}_{0}\right]$. As a result, the dispersive resonator suppresses the bandwidth with the scaling factor of $\frac{\Delta {\omega }_{{\rm{d}}}}{\Delta {\omega }_{{\rm{nd}}}}=\frac{n}{n+D}$.

This method was initially used in FP optical cavities to suppress the output linewidth of lasers, where the dispersive media was created by additional pumping the cesium atomics using the effect of coherent population trapping^40^. Recent research has demonstrated that the mode supported by nanoresonators such as metasurfaces or gratings have large dispersion near the resonant frequency^41^, where the phase of the reflection is linearly dependent on the frequency. Therefore, by combing a metasurface with a FP cavity (e.g., a dielectric film), it is possible to suppress the linewidth of the FP resonance. This strategy has been adopted to produce all-dielectric high-Q absorbers^42^.

#### Material-assisted high-Q resonances

Some special materials such as superconducting materials and gain materials can help to reduce the non-radiative decay rates, and thereby increase the non-radiative Q-factors of modes, see Fig. 3g. Superconducting materials exhibit lower real conductivity and higher imaginary conductivity compared to ordinary conductors. This characteristic results in a lower loss tangent, enabling the modes to achieve higher Q-factors. For instance, yttrium barium copper oxide (YBCO) metausrfaces, below their phase transition temperature (87 K), can support Fano resonances in the THz range with higher Q-factors compared to the same structures made from aluminum^43^. Gain materials have the ability to compensate for the intrinsic Ohmic losses in metallic elements, thereby reducing the non-radiative decay rate^44^. This compensation effect helps to preserve the energy of the modes, resulting in smaller losses and higher non-radiative Q-factors.

## Progress

Based on the working purposes, free-space high-Q nanophotonic devices can be categorized into those of non-absorbing type and absorbing type. The former usually acts as a high-Q reflector or band-pass filter, which mainly focuses on engineering the radiative loss while the non-radiative loss should be minimized. The latter usually acts as a high-Q absorber or thermal emitter, where the non-radiative loss is necessary and should be engineered simultaneously with the radiative loss to achieve efficient absorption.

### High-Q non-absorbing devices

**Fig. 4:**
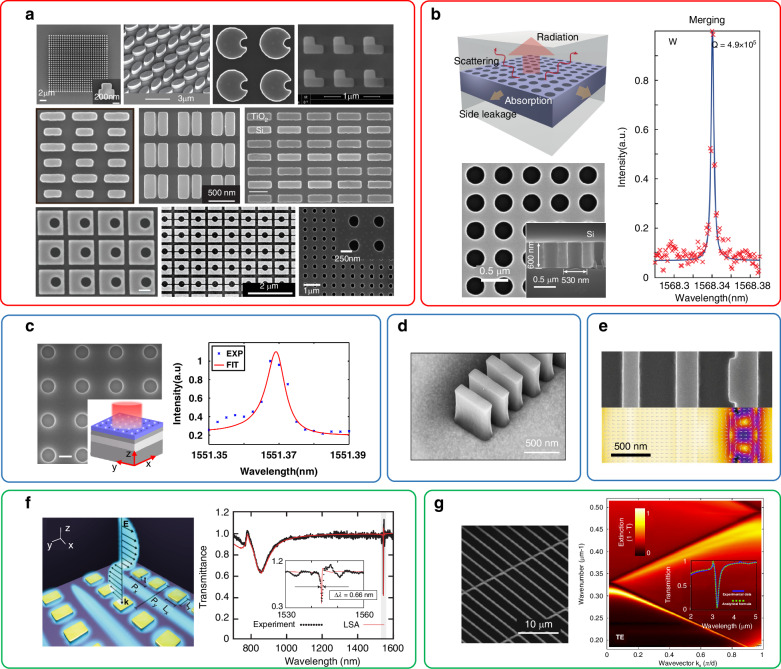
High-Q non-absorbing nanophotonic devices. **a** Various nanostructures supporting qBICs^4,30,45–52^. **b** PhCs with multiple BICs merging at the same point in the momentum space^12^. **c** GMRs based on PhCs^53^. **d** GMRs based on periodic nanoantenna arrays^55^. **e** GMRs combined with phase-gradient metausrfaces^56^. **f** SLRs based on metallic metasurfaces^57^. **g** SLRs based on dielectric nanostructures^58^. Panels adapted from: AAAS^4^; ACS^30,46,47,49,52^; Springer Nature^12,48,53,55–57^; APS^45,58^; Wiley^50^; De Gruyter^51^

In non-absorbing nanophotonic devices, high-Q transmission or reflection can be achieved utilizing optical modes with small radiative decay rates and negligible non-radiative decay rates. A widely adopted approach involves the utilization of lossless nanostructures supporting BICs such as all-dielectric metasurfaces^4,30,45–52^, as shown in Fig. 4a. In principle, optical modes with infinite Q-factors can exist in these nanostructures due to the non-radiative nature of ideal BICs and the non-absorbing property of dielectric materials. However, to enable the excitation of these modes by external light, a small geometric perturbation is introduced. This modification transforms BICs into qBICs, which exhibit finite but extremely high Q-factors. In experiments, the Q-factors may be degraded due to the finite size of the nanostructure arrays and various fabrication imperfections. Nevertherless, a Q-factor of 4.9 × 10^5^ has been experimentally demonstrated by merging multiple BICs supported by photonic crystals (PhCs) in momentum space^12^, see Fig. 4b.

Another commonly used type of mode is GMRs. An experimental Q-factor as high as 2.39 × 10^5^ has been demonstrated recently using a photoresist grating on silicon-on-insulator (SOI)^53^, see Fig. 4c. Using similar methods, a recent study demonstrated a Q-factor exceeding one million by employing photoresist gratings on silicon nitride (SiN)^54^. In addition to conventional 1D or 2D gratings, many new types of gratings with special geometry and specific functions have been designed. In ref. ^55^ periodic gratings composed of isolated nanoantennas are utilized, resulting in a strong electric field enhancement within the air gap for biosensing applications, see Fig. 4d. In ref. ^56^ gratings are combined with phase-gradient metasurfaces to achieve high-Q directional diffraction, as shown in Fig. 4e.

SLRs can also be employed to achieve high-Q non-absorbing devices. most of the works are based on periodic metallic nanostructures to demonstrate enhanced Q-factors in comparison to localized surface plasmons supported by individual metallic nanoparticles. Despite the intrinsic loss of metals, a Q-factor of 2340 has been demonstrated in metallic metasurfaces supporting SLRs^57^, as illustrated in Fig. 4f. SLRs have also been achieved in dielectric photonic systems, where the radiative Q-factor of a monolayer of dielectric nanorods is increased within a lattice array^58^, see Fig. 4g.

In addition to the aforementioned methods, there are other approaches to achieve high-Q non-absorbing photonic devices. One such method involves utilizing high-order Mie resonances, where the Q-factor can reach 1475 in experiment^36^. Another approach involves utilizing a pair of coupled bright and dark modes, similar to the EIT effect, to achieve high-Q transmission, where the Q-factor is 483^21^. Table 1 summarizes the high-Q non-absorbing devices based on different design strategies for comparison^59–69^.

**Table 1:** High-Q non-absorbing devices

Mode	Q (exp.)	λ (μm)	Structure	Efficiency^a^	Polarization Selective	Size	Ref.
BICs	490,000	1.568	Si PhCs	(S)	No	250 × 250 μm²	^12^
	~10,000	0.583	Si_3_N_4_ PhCs	44% (R)	Yes	3 cm²	^59^
	7800	1.56	Si PhCs	(S)	No	500 × 500 μm²	^60^
	4990	1.479	Si metasurfaces	(R)	Yes	Unmentioned	^50^
	18,511	1.559	Si metasurfaces	(T)	Yes	19 × 19 μm²	^45^
	3534	1.548	Si metasurfaces	60% (T)	Yes	120 × 120 μm²	^61^
	3142	1.48	Si metasurfaces	55% (S)	Yes	15 × 15 mm²	^62^
	1946	1.425	Si metasurfaces	(S)	Yes	410 × 410 μm²	^46^
	1373	1.275	Si metasurfaces	45% (T)	Yes	560 × 560 μm²	^63^
	1011	1.28	Si metasurfaces	(S)	Yes	280 × 280 μm²	^52^
	4130	1.52	Si metasurfaces	(T)	Yes	1.2 × 1.2 mm²	^64^
GMRs	1,100,000	779	Photoresist PhCs on SiN	(R)	Yes	900 × 900 μm²	^54^
	239,000	1.55	Photoresist PhCs on Si	(R)	Yes	520 × 520 μm²	^53^
	32,000	0.49	Si_3_N_4_ PhCs	34% (R)	Yes	600 × 600 μm²	^65^
	8000	0.75	SiO_2_ gratings on a SiN film	(R)	Yes	10 × 15 mm²	^66^
	3238	1.583	SiO_2_-Si crossed gratings	53% (R)	No	4 cm²	^67^
	2500	1.465	Si gratings	20% (D)	Yes	300 × 300 μm²	^56^
	2200	1.53	Si blocks	(T)	Yes	Unmentioned	^55^
SLRs	2340	1.55	Au metasurfaces	56% (S)	Yes	600 × 600 μm²	^57^
	750	1.097	Al metasurfaces	40% (S)	Yes	Unmentioned	^68^
High-order modes	1458	1.293	Si metasurfaces	17.1% (D)	Yes	Unmentioned	^36^
	728	1.508	Si metasurfaces	(T)	No	90 × 90 μm²	^69^
Others	483	1.372	Si metasurfaces	60% (T)	Yes	225 × 225 μm²	^21^

^a^S, D, T, and R in the table represent scattering, diffraction, transmission and reflection efficiency, respectively

### High-Q absorbing/ thermal emitting devices

In contrast to non-absorbing photonic devices discussed in Section “High-Q non-absorbing devices”, whose non-radiative decay rates should be minimized, absorbing devices require careful management of non-radiative decay rates so as to absorb light efficiently. Achieving high-Q efficient absorption involves two key considerations. Firstly, the radiative and non-radiative decay rates should be simultaneously reduced to ensure a large overall Q-factor. Secondly, these two decay rates should be equal to each other to satisfy the critical coupling condition, enabling efficient absorptance. The most commonly employed methods for achieving high-Q absorbing nanophotonic devices are BICs, GMRs, and Tamm plasmon polaritons.

Absorption and thermal emission are two closely related physical processes according to Kirchhoff’s law, which states that the thermal emissivity *ε*(*λ*) of an object equals to its absorptance *A*(*λ*) in reciprocal systems^70^. Therefore, a nanophotonic device with high-Q absorption can also exhibits high-Q thermal emissivity, and can have high-Q efficient thermal emission when the absorption peak is at the mid-infrared range.

**Fig. 5:**
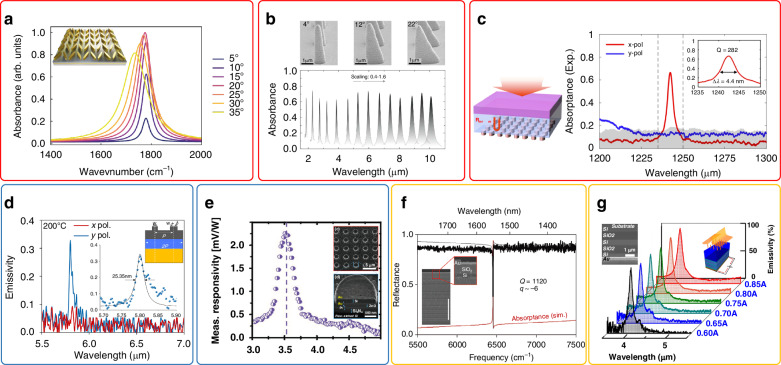
High-Q absorbing/ thermal emitting nanophotonic devices based on BICs, GMRs, and Tamm plasmon polaritons. **a** Plasmonic BICs based on MIM structures^71^. **b** Plasmonic BICs based on anisotropic metasurfaces^72^. **c** High-Q absorbers based on dielectric metamirror with dispersive reflection^42^. **d** High-Q thermal emission based on 1D GMRs^78^. **e** High-Q absorption based on 2D GMRs^79^. **f** High-Q absorption based on Tamm plasmon polaritons^80^. **g** High-Q thermal emission based on Tamm plasmon polaritons^81^. Panels adapted from: ACS^42,81^; Wiley^71,79,80^; AAAS^72^; APS^78^

For high-Q absorption based on BICs, in order to introduce non-radiative loss, one straightforward method is to replace dielectric materials with metals in nanostructures that supports BICs, resulting in plasmonic BICs^71^, see Fig. 5a. With the development of laser nanoprinting techniques, plasmonic BICs have been experimentally achieved using 3D anisotropic metasurfaces^72,73^, as shown in Fig. 5b. However, due to the intrinsic losses of metals, the Q-factors based on plasmonic BICs in experiments are typically less than 100. Recent theoretical research reveals that the achievable maximum Q-factor for plasmonic BICs has inherent limits approaching that of propagating SPPs, and increases with the operating wavelength^74^. Besides the metallic structures, low-loss dielectric metasurfaces supporting BICs can also be used to realize high-Q absorption^75,76^. As the total Q-factor of an absorber is given by ${{Q}_{{\rm{tot}}}}^{-1}={{Q}_{{\rm{r}}}}^{-1}+{{Q}_{{\rm{n}}}}^{-1}$, the *Q*_tot_ is anticipated to be always lower than *Q*_r_ in single-mode systems. To overcome this limitation, in ref. ^42^ dielectric metasurfaces supporting BICs are used as the dispersive reflector in a FP cavity. This dispersion-assisted FP mode enables the total Q-factor of the absorber to exceed that of the BIC itself. In experiment, a Q-factor of 282 is demonstrated, see Fig. 5c.

For high-Q absorption based on GMRs, it can be achieved by introducing periodic perturbations to the surfaces that support lossy guided modes such as SPPs or surface photon polaritons (SPhPs)^77^. Besides, GMRs can also be supported by dielectric waveguiding films with gratings on top, where the dispersion properties of the guided modes can be engineered by changing the thickness of the films. The polarization response can be engineered through the design of the top gratings. For instance, 1D gratings enables polarization-sensitive high-Q absorption with the Q-factor of 230^78^, see Fig. 5d. While 2D gratings with isotropy in both the x and y directions enable polarization-insensitive absorption with the Q-factor of 73^79^, see Fig. 5e.

In addition to BICs and GMRs, another type of mode frequently utilized in high-Q absorption or thermal emission design is Tamm plasmon polaritons. Experimental studies have demonstrated that absorption based on Tamm plasmon polaritons can have Q-factors in the order of 10^3^ ^80^, surpassing previous metasurfaces of gratings designs, see Fig. 5(f). Besides, different from planner metasurfaces or gratings that require precise and time-consuming fabrication techniques, the multi-layer film structures used in Tamm plasmon polaritons can be easily fabricated using conventional deposition methods such as sputtering, electron beam evaporation, and thermal evaporation. This makes them well-suited for applications requiring large-area fabrications such as thermal emitters^81^, see Fig. 5g. However, it is worth noting that Tamm plasmon polaritons lack the ability for polarization and directionality control, which can be considered as their limitations for applications.

**Fig. 6:**
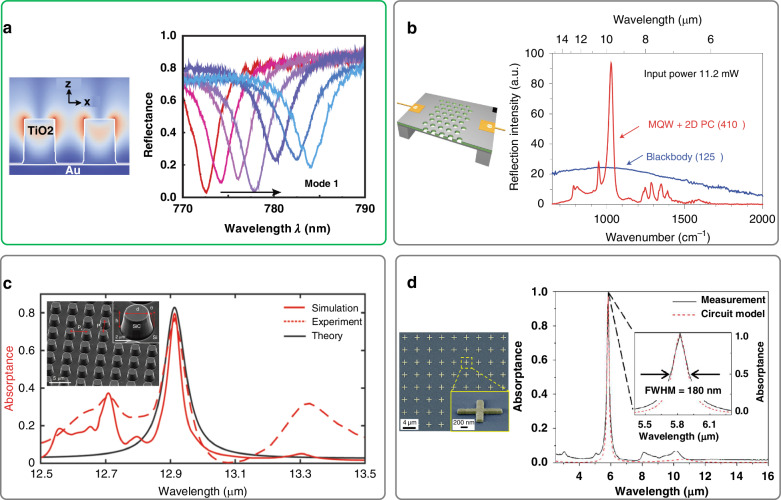
High-Q absorbing/ thermal emitting nanophotonic devices based on SLRs, PhCs, high-index dielectric metasurfaces, and MIM structures. **a** SLRs composed of TiO2 metasurfaces^82^. **b** PhCs combined with multiple quantum wells^83^. **c** SiC metasurfaces with a high refractive-index near the SPhPs resonance^84^. **d** MIM absorbing metasurfaces^86^. Panels adapted from: Wiley^82,84,86^; Springer Nature^83^

Alternative methods for achieving high-Q absorption or thermal emission have also been explored. Reference ^82^ utilizes SLRs supported by low-index TiO_2_ metasurfaces on Au substrate to achieve high-Q absorption, where the Q-factor in experiment is larger than 200, as depicted in Fig. 6a. Reference ^83^ combines PhCs with multiple quantum wells to achieve high-Q thermal emission. The multiple quantum wells enable the generation of narrowband absorption through intersubband transitions. When this narrowband absorption matches with the high-Q mode supported by the PhCs, a high-Q thermal emission with the Q-factor of around 30 can be generated, see Fig. 6b. Reference ^84^ uses high-index dielectric metasurfaces to achieve high-Q thermal emission. By employing SiC, which possesses a high refractive index (~10) at the low-frequency side of its surface phonon polariton resonance, the metasurfaces can support ED and MD with higher Q-factors compared to conventional semiconductors such as Si and Ge, as shown in Fig. 6c, where the Q-factor of the thermal emission is 170.

Additionally, it is worth noting that metal-insulator-metal (MIM) structures that support localized SPPs are commonly employed in the design of selective absorbers, spanning from visible light to microwaves^85^. However, the localized nature of these modes and the large absorption losses in the metals often restrict the Q-factors to be less than 10. A recent study has shown that MIM structures supporting localized SPPs exhibit a maximum Q-factor of approximately 33^86^, as depicted in Fig. 6d. A summary of the high-Q absorbing or thermal emitting devices utilizing various design strategies mentioned above is presented in Table 2^87–95^.

**Table 2:** High-Q absorbing/ thermal emitting devices

Mode	Q (exp.)	λ (μm)	Structure	Efficiency^a^	Polarization selective	Size	Ref.
BICs	120	9.1	GaAs-AlGaAs PhCs	71% (E)	No	1.8 × 1.8 mm²	^87^
	282	1.237	Ge-SiO_2_-Si metasurfaces	66.5% (A)	Yes	120 × 120 μm²	^42^
	84	1.8-10	Au-resist metasurfaces	60% (A)	Yes	160 × 160 μm²	^72^
	81	6.7	Ge metasurfaces-Al_2_O_3_-Au	30% (E)	Yes	>50 × 50 μm²	^88^
	45	6	Au metasurfaces-CaF_2_-Au	80% (A)	Yes	115 × 115 μm²	^71^
	66	0.832	Ag metasurfaces-Al_2_O_3_-Ag	35% (A)	Yes	Unmentioned	^89^
GMRs	72	9.2	GaAs PhCs	74% (E)	No	1.8 × 1.8 mm²	^9^
	229	5.78	Ge gratings-Al2O3-Au	35% (E)	Yes	Unmentioned	^78^
	118	3.532	W gratings	55% (E)	No	>100 × 100 μm²	^90^
	100	11.36	SiC gratings	94% (E)	Yes	50 × 50 μm²	^77^
	73	3.722	Au metasurfaces	99% (A)	No	2 × 2 mm²	^79^
	180	0.8	Si metasurfaces	22% (A)	Yes	43 × 37 μm²	^8^
	101	10.9	Ge gratings-SiC	84% (E)	Yes	0.8 × 0.8 cm²	^71^
SLRs	300	0.77-0.9	TiO_2_ metasurfaces-Au	93% (A)	Yes	Unmentioned	^82^
	63	0.75	Au metasurfaces	90% (A)	No	300 × 300 μm²	^92^
Tamm plasmon polaritons	1120	1.55	Au-DBR (Si/ SiO_2_)	92% (A)	No	/	^80^
	780	4.57	DBR (Ge/ SiO_2_)-Pt	36% (E)	No	/	^93^
	172	4.1	cGST-DBR (Ge/ ZnS)-ZnS-Ge-Au	65% (E)	No	/	^94^
	88	4.731	DBR (Ge/ SiO2)-SiO_2_-Au	90% (E)	No	/	^95^
	170	12.9	SiC metasurfaces	78% (A)	No	Unmentioned	^84^
	164	10	hBN/α-MoO3-Au	36% (E)	Yes	≤200 × 200 μm²	^79^
	33	5.83	Au metasurfaces-SiO_2_-Au	99.7% (A)	No	100 × 100 μm²	^86^

^a^E and A in the table represent thermal emission and absorption efficiency, respectively

## Applications

With the fast development of nanofabrication technologies, free-space high-Q nanophotonics have found applications in numerous fields. For high-Q non-absorbing devices with a narrowband reflection or transmission spectrum, they have been used in the fields such as sensing, filtering, lasing, and nonlinear optics. For high-Q absorbing devices with a narrowband absorption spectrum, they are typically used in the field of photodetection, coherent thermal emission, and laser stealth.

### Sensing

Optical sensing technology provides an efficient way to detect the targets by analyzing the light signals. A widely used strategy for sensing is based on detecting the resonance shift of modes caused by the change of refractive index in the surrounding environment. The parameter to evaluate the sensing performance is figure-of-merit (FOM). It is calculated as ${\rm{FOM}}=S/\Delta \lambda$, where *S* is the sensitivity, defined as the wavelength shift per refractive index unit (RIU), and $\Delta \lambda$ is the bandwidth of the resonance. A superior sensor with a high FOM requires simultaneous high sensitivity and narrow resonance bandwidth, which can be satisfied using high-Q modes. On the one hand, a high-Q mode has a narrower bandwidth $\Delta \lambda$ compared with a low-Q one. On the other hand, a high-Q mode can have stronger near-field enhancement since the enhancement coefficient *P* can be evaluated as: $P\propto {\gamma }_{{\rm{r}}}{Q}^{2}/V$, where *γ*_r_ is the radiative decay rate and *V* is the mode volume^96^. A large field enhancement can enhance the light-mater interactions and improve the sensitivity *S*. Various high-Q optical sensors with superior sensing abilities have been proposed. For instance, refractive index sensing with the FOM of 103 has been experimentally demonstrated using Fano-type resonances with the Q-factor of 483^21^. Genetic screening with the FOM as high as 400 has been achieved using GMRs with the Q-factor of 2200^55^.

Another strategy for optical sensing is based on detecting the absorption spectra of analytes, where multiple high-Q modes with different resonant frequencies are integrated together to function as a spectrometer. This strategy allows for distinguishing the chemical information of the analytes. For instance, mid-infrared sensing for molecular barcoding has been demonstrated based on qBICs with the Q-factors larger than 200^4^. Near-infrared biodetection combined with hyperspectral imaging has been achieved using qBICs with the Q-factors of 144^97^.

### Filters

High-Q nanophotonic devices with narrow bandwidths allow for filtering specific wavelengths and suppressing unwanted wavelengths as well as background noise. These optical filters with high spectral selectivity have been applied in numerous fields ranging from spectrometers to imaging systems. For example, traditional spectrometers require dispersive elements such as prisms or gratings to detect the spectra, making the devices bulky and require precise collimation. High-Q filters offer an alternative way to obtain the spectra intensity without any dispersive elements. Their planarity and small footprint promote the miniaturization of spectrometers^98^. In imaging systems, high-Q filters can be employed to control the color reproduction and improve the contrast ratios. Besides, they can also help to resolve the wavelength information from snapshot imaging data, enabling the development of hyperspectral imaging techniques in visible range^99^.

High-Q filters with compact size, tunable wavelength, and high efficiency have been demonstrated using plasmonic^100,101^ and dielectric^102^ metasurfaces, where the maximum Q-factor can approach 10^3^. However, their main disadvantage is that large-scale fabrication is expensive and time consuming. To tackle this problem, grayscale photolithography has been employed to fabricate wafer-scale filter arrays based on FP resonances. The cavity thickness can be adjusted by varying the photolithography exposure dose, allowing for FP resonant wavelengths span the visible light range^5^.

### Lasing

High-Q optical modes with the ability to trap and enhance electromagnetic fields can help to enhance the light-matter interactions in the resonator, which has been demonstrated to facilitate the strong coupling of excitons with photons in semiconductor materials^103^, and helpful for directional emission^104^^,^^105^. In lasing systems, the enhanced light-matter interactions can lead to a low pumping threshold. For example, utilizing the qBICs supported by GaAs nanoantenna arrays, directional lasing with a Q-factor of 2750 can be achieved, whose pumping threshold is ~14 μJcm^−2^^105^. Song et al. demonstrate that for perovskite metasurfaces supporting BICs, lasing with a Q-factor larger than 550 can happen with the pumping threshold as low as ~4.2 μJcm^−2^. Moreover, utilizing the far-field properties of BICs, the output laser can be tuned from vortex beams to linearly polarized beams with a switching time as fast as 1.5 picoseconds^6^.

Besides the large field enhancement, high-Q nanostructures such as metasurfaces and gratings can be integrated directly onto a flat substrate, enabling miniaturization and integration of the lasing systems. Recent work has demonstrated that lasing action with a Q-factor of ~4700 can happen in BIC cavities with 16-by-16 nanoresonators. By increasing the pump power, the number of resonators can be scaled down to as few as 8-by-8 nanoresonators, opening pathways to miniaturization of lasing devices^106^.

### Nonlinear optics

Nonlinearity is the phenomena where the optical properties of materials change with the intensity of light, leading to a nonlinear relationship between the incident light and the induced effects. High-Q nanophotonic devices have shown great advantages in the exploration of new physics and applications in nonlinear optics. On the one hand, the strong resonances of nanoresonators can relax the momentum-matching requirements between the pump and generated signals. On the other hand, the large near-field enhancement can help to boost the nonlinear responses, which are otherwise inherently weak and necessitate large bulk volumes in natural materials. These advantages enable the devices exhibit strong nonlinear optical responses in a much more compact footprint^107^.

For example, owing to the strong field enhancements, the sum-frequency generation efficiency in high-Q BIC-type metasurfaces can be two orders of magnitude higher than that in low-Q Mie-type metasurfaces^108^. Combined with Si metasurfaces supporting BICs, the second-harmonic intensity of a WS_2_ monolayer can be enhanced by more than 3 orders of magnitude^109^. In multilayer photonic-band-gap structures, the density of mode can be enhanced at the band edge^110^. This can increase the light-mater-interaction time, and thereby enhance the nonlinearity. Utilizing multilayer photonic-band-gap structures, the efficiency of second-harmonic generation can be enhanced by a factor of 30 with respect to that from a single Ag film^111^. Other nonlinear effects such as third-harmonic generation^45^, and spontaneous parametric down-conversion^7^ have also been demonstrated using high-Q nanophotonic devices, which can be used for wavefront shaping^112^ and complex quantum states generating^7^.

### Photodetection

Photodetection is based on the absorption of photons to generate a measurable photocurrent, whose magnitude represents the intensity of the incident light. Traditional photodetection methods based on the intrinsic absorption of bulk materials generally have low efficiencies. In comparison, high-Q nanophotonic devices can enhance the light-matter interactions due to the strong field enhancement, leading to the increase of absorption and improved sensitivity in detecting weak light signals. For example, Ge metasurfaces with a Q factor of ~15 can increase the photocurrent by 5 times compared with that of unpatterned Ge films near 1550 nm^113^. Besides, due to the enhanced light absorption in nanostructures, the required thickness of the absorbing materials in photodetectors can be far less than the working wavelength. For example, subwavelength-thick Si metasurfaces supporting high-Q nonlocal modes (Q ~ 80) can produce significant optical absorption (50%) despite the weak material absorption of Si near 800 nm^8^. The thin thickness of high-Q nanophotonic devices allows for fast response time and are promising for high-speed photodetection.

### Coherent thermal emission

Thermal emission is incoherent by nature, which is broadband and omnidirectional. The radiance of an object with the emissivity of *ε*(*λ*) at the temperature *T* is given by Plank’s law

10 $$I\left(\lambda \right)=\varepsilon \left(\lambda \right)\,\cdot\, \frac{2h{c}^{2}}{{\lambda }^{5}}\,\cdot\, \frac{1}{{e}^{\frac{{hc}}{\lambda {k}_{B}T}}-1}$$

where *λ* is the wavelength, *h* is Plank’s constant, *c* is the vacuum light speed, *kB* is the Boltzmann constant. As has been introduced in Section “High-Q absorbing/ thermal emitting devices”, Kirchhoff’s law equates emissivity $\varepsilon \left(\lambda \right)$ to absorptance $A\left(\lambda \right)$. Therefore, nanophotonic devices with high-Q absorption enable the thermal emission to happen within a narrow bandwidth, allowing for increasing the coherence of thermal sources at targeted wavelengths.

**Fig. 7:**
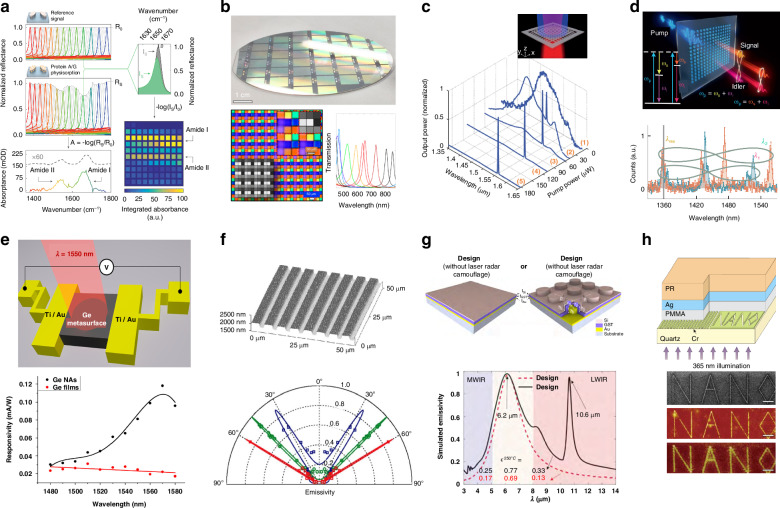
Applications of free-space high-Q nanophotonic devices. **a** High-Q metasurfaces for biosensing^4^. **b** High-Q optical filters based on FP resonances^5^. **c** High-Q metasurfaces for lasing^106^. **d** High-Q nonlinear metasurfaces for generating complex quantum states^7^. **e** High-Q Ge absorbers for photodetection^113^. **f** High-Q SiC gratings for narrowband thermal emission^77^. **g** High-Q gratings for laser stealth^10^. **h** High-Q GMRs for super resolution imaging^122^. Panels adapted from: AAAS^4,7,122^; ACS^5,113^; Elsevier^10^; Springer Nature^77,106^

Coherent thermal sources have been realized using high-Q absorbers such as 1D gratings^77^ (Fig. 7f), 2D photonic crystals^114^, metasurfaces^115,116^, and film stacks^81,117,118^. From Table 2, it can be seen that the Q-factors of thermal emitters based on nanostructures such as metasurfaces and gratings are typically less than 300, while those based on film stacks can be more than 10^3^ since there is no need to process complex nanostructures. It should also be noted that not all absorbers can act as thermal emitters as the thermal emission power is also dependent on the temperature from Plank’s law. At the room temperature of 300 K, the peak of blackbody thermal emission power is at around 10 μm. Therefore, the high-Q absorbers should have the resonance wavelength at infrared range and can withstand high temperature so as to act as high-Q thermal emitters.

### Laser stealth

Laser stealth is the technique that enables targets to absorb or scatter the incident light at the corresponding wavelengths so that no reflecting signals from the targets can be detected^119^. Although broadband absorbers can achieve stealth at multiple wavelengths, they are difficult to be compatible with other functions since light within a broad wavelength range is absorbed. In comparison, high Q absorbers can selectively absorb light at a specific wavelength and transmit or reflect the light at other wavelengths, which can be applied to stealth devices that are compatible with camouflage, radiative cooling, and radiative warming. For example, infrared thermal camouflage devices with CO_2_ laser stealth devices have been demonstrated, which have high reflection over the whole atmosphere transparent window (8–14μm) except at the wavelength of 10.6μm due to the high-Q absorption of SLRs^10^ (Fig. 7g).

### Super resolution imaging

Super-resolution imaging is a technique which can enhance the resolution beyond the diffraction limit, allowing for the visualization of details much smaller than the illumination wavelength. It can be achieved by amplifying evanescent waves that carry subwavelength information about the object^120,121^. Propagating guided waves such as SPPs in metal films and GMRs in dielectric slabs can be utilized to amplify the evanescent waves. For example, based on the SPPs modes in propagating along the surface of Ag films, super resolution for p-polarized illumination have been demonstrated in the visible range, whose resolution is on the order of one-sixth of the illumination wavelength^122^. Based on the waveguide mode in GaAs dielectric layers, super resolution for s-polarized illumination can also be achieved^123^.

## Conclusions and outlook

**Fig. 8:**
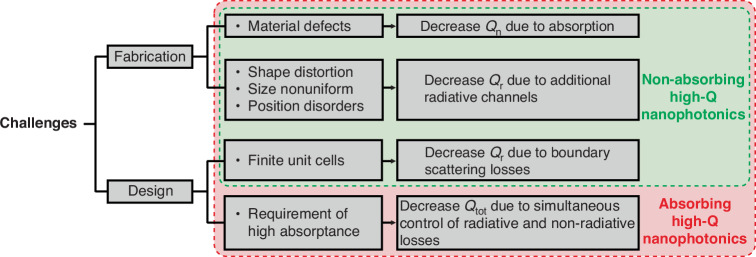
Challenges on increasing the Q-factors of free-space high-Q nanophotonic devices

In this Review, we provide a comprehensive summary on the physical principles, recent advancements, and the diverse applications of free-space high-Q nanophotonics. Due to their nontrivial properties and high integration, high-Q nanophotonic devices have opened up new avenues in physics, applied science, and biochemistry. Although numerous achievements have been made in achieving high Q-factors experimentally, there still exist challenges remaining to be addressed to further increase the Q-factors for non-absorbing and absorbing devices, as summarized in Fig. 8.

For non-absorbing devices, the degradation of the total Q-factors in experiments can result either from additional non-radiative loss or radiative loss. The former can arise from material absorption due to the fabrication defects, while the latter can arise from multiple aspects. For example, the finite number of units in periodic structures can introduce additional scattering losses at the boundaries^124^. Besides, the fabrication imperfections such as shape, size, and position disorders can impart extra momentum to the mode, leading to the coupling of modes at different points in k-space and the creation of additional radiative channels. In order to achieve high Q-factors experimentally, merging of multiple BICs at the same point can be utilized^12^. Contrasted with a single isolated BICs^125–127^, slight deviations in the scattering wavevectors resulting from fabrication imperfections have a diminished influence on the Q-factors of merging BICs. This phenomenon ascribes to the fact that merging BICs enhances the Q-factor of all waves possessing closely aligned wavevectors with the resonance.

Furthermore, for free-space devices such as metasurfaces or gratings, the number of unit cells becomes a crucial consideration for practical applications. Typically, the Q-factors tend to increase with the number of units^45^, reaching a maximum for infinite arrays due to the suppression of scattering losses at the boundaries. However, the fabrication imperfections also increase with the number of units, which can consequently decrease the Q-factors. Therefore, a trade-off should be found in experiments to attain high Q-factors.

For absorbing devices, the design requirements become more stringent as they necessitate not only high Q-factors but also high absorptance. Therefore, in addition to reducing radiative and non-radiative losses, it is also crucial to independently engineer these two types of losses to reach the critical coupling condition. A comparison between high-Q non-absorbing and absorbing free-space devices reveals that Q-factors on the order of 10^5^ have been experimentally achieved in non-absorbing devices, while absorbing devices are typically limited to Q-factors of 10^3^. Advanced strategies are currently required to better engineer the radiative and non-radiative Q-factors independently so as to obtain high-Q absorbers with high absorbing efficiency.

As a prospect for this field, while in review we mainly focus on achieving high Q-factors utilizing periodic nanostructures, it is worthwhile to explore how to increase the Q-factors in isolated nano-antennas^128^. Single nano-antenna excited by free-space can support resonances similar to those in periodic metasurfaces^129^. Increasing the Q-factors offers the potential for enhancing the light-mater interactions in a single nano-antenna, which can be applied in fields such as far-field subwavelength position detection^130^.

Besides, we note that while most high-Q nanophotonic devices have focused on spectral control to achieve narrowband responses, the importance of wavevector control is sometimes overlooked. Recent works have shown possibilities for engineering the wavevectors of high-Q nanophotonic devices. For instance, ref. ^131^ demonstrated the control of k-space dispersion by combining a grating with a planar cavity; ref. ^132^ demonstrated that high-Q modes with flat band dispersions can be achieved by halving the first Brillouin zone; ref. ^56^ showcased the output wavefront of high-Q modes can be controlled using a combination of nonlocal guided modes and local Mie resonances. The ability to manipulate the wavevector is particularly significant in free-space applications like lasing and wireless communications.

Recently, research on high-Q nonlocal metasurfaces has been gradually attracting more attention and interest. These metasurfaces exhibit a response characteristic where the behavior at a specific point is influenced not only by the local fields at that point but also by the fields at neighboring points^133^. This nonlocality enables the metasurfaces to achieve wavevector-dependent control of scattering amplitudes, thereby allowing for enriched optical responses. They have found applications in various fields such as AR/VR devices^134^, wavelength-selective Huygens’ metalens^135^, customized thermal metasurfaces^136^, customized radiation of leaky waves^137^, and imaging^138^. We anticipate that further exploration of simultaneously engineering the spectral and wavevector properties of high-Q modes will unlock a wide range of new functionalities in nanophotonic devices.
